# Draft Genome Sequence of *Chromobacterium aquaticum* CC-SEYA-1, a Nonpigmented Member of the Genus *Chromobacterium*

**DOI:** 10.1128/genomeA.01661-16

**Published:** 2017-03-23

**Authors:** Scott D. Soby

**Affiliations:** Biomedical Sciences Program, College of Health Sciences and College of Veterinary Medicine, Midwestern University, Glendale, Arizona, USA

## Abstract

*Chromobacterium aquaticum* CC-SEYA-1^T^, isolated from a spring in Taiwan, shares many characteristics with other members of the genus but also contains auxin biosynthesis genes and does not produce the pigment violacein. *Chromobacterium* sp. 49, isolated from Brazil, is identified here as *C. aquaticum*, indicating that this is a cosmopolitan species.

## GENOME ANNOUNCEMENT

*Chromobacterium aquaticum* CC-SEYA-1T is a nonproducer of the purple pigment violacein and was isolated from a mountain spring on the island of Taipei. It was recognized as a new species in 2008 (1). Although there has been some additional information published about this organism, its environmental role is unclear (2), and there are no reports of pathogenesis associated with this bacterium. The genus *Chromobacterium* has undergone a rapid expansion since 2007 (3–8), and the completion of a collection of genomic sequences of all of the species with standing in the literature will be important in redefining or refining the genus. The genome of *C. aquaticum* CC-SEYA-1 was sequenced at the Arizona State University CLAS Genomics Core facility using Illumina MiSeq. Genomic DNA was sheared to approximately 600-bp fragments using a Covaris M220 ultrasonicator, and Illumina libraries were generated on an Apollo 384 liquid handler (Wafergen) using a Kapa Biosystems library preparation kit (catalog no. KK8201). DNA fragments were end-repaired and A-tailed as described in the Kapa protocol. Combined indexes/adapters (catalog no. 520999; Bioo) were ligated onto each sample and multiplexed into one lane. Adapter-ligated molecules were cleaned using AMPure beads (catalog no. A63883; Agencourt Bioscience/Beckman Coulter, Inc.) and amplified with Kapa HIFI enzyme. Libraries were analyzed on an Agilent Bioanalyzer and quantified by quantitative PCR (qPCR) (catalog no. KK4835; Kapa library quantification kit) before multiplex pooling and sequencing in a 2 × 300 paired-end (PE) flow cell on the MiSeq platform (Illumina). Adapters were computationally segregated and trimmed in the Illumina BaseSpace pipeline. The Velvet assembly tool (BaseSpace) was used for signal processing and partial sequence assembly. The sequence is 63.51% G+C and consists of 4,997,664 bp distributed over 171 scaffolds (≥0 bp), 117 of which are larger than 1 kbp. The largest contig is 311,811 bp, the *N*_50_ is 88,237 bp, and the *N*_75_ is 44,928 bp, with a sequence coverage of 42.03×.

*Ab initio* gene prediction was performed on the assembly using RAST (http://rast.nmpdr.org/). There are 4,434 predicted genes in the genome, only 48% of which are identifiable in the RAST/SEED servers. Like many of the other *Chromobacterium* spp., the *C. aquaticum* genome contains homologs to *Mycobacterium* virulence operons for protein synthesis, DNA transcription, and quinolinate biosynthesis, siderophores, heme uptake, chitinases, and *N*-acetylglucosamine transport pathways. Unlike other *Chromobacterium* spp., the genome contains genes for auxin biosynthesis via the indole-3-acetaldehyde pathway and an AUX1-like permease. Genes are present for the synthesis of enterobactin siderophores, cyanate hydrolysis, lysozyme inhibitors, and heme/hemin uptake systems. The *C. aquaticum* CC-SEYA-1 genome sequence was compared to reference genomes of *Chromobacterium violaceum*, *Chromobacterium subtsugae*, *Chromobacterium haemolyticum*, *Chromobacterium vaccinii*, *Chromobacterium piscinae*, *Chromobacterium pseudoviolaceum*, and *Chromobacterium* sp. LK1, LK11, and 49, using the Genome-to-Genome Distance Calculator (GGDC) (9, 10). The *C. aquaticum* genome is 93.9% homologous with *Chromobacterium* sp. 49 (11) but less than 30% homologous to the other reference genomes. *Chromobacterium* sp. 49 thus is an isolate of *C. aquaticum*.

### Accession number(s).

This whole-genome shotgun project has been deposited at DDBJ/EMBL/GenBank under the accession number MQZY00000000. The version described in this paper is version MQZY01000000.

## References

[1] Young CC, Arun AB, Lai WA, Chen WM, Chou JH, Chao JH, Shen FT, Rekha PD, Kämpfer P 2008 *Chromobacterium aquaticum* sp. nov., isolated from spring water samples. Int J Syst Evol Microbiol 58:877–880. doi:10.1099/ijs.0.65573-0. 18398186

[2] Rekha PD, Young CC, Arun AB 2011 Identification of *N*-acyl-l-homoserine lactones produced by non-pigmented *Chromobacterium aquaticum* CC-SEYA-1 and pigmented *Chromobacterium subtsugae* PRAA4-1. 3 Biotech 1:239–245. 10.1007/s13205-011-0029-1 PMC3339584 22558542

[3] Zhou S, Guo X, Wang H, Kong D, Wang Y, Zhu J, Dong W, He M, Hu G, Zhao B, Zhao B, Ruan Z 2016 *Chromobacterium rhizoryzae* sp. nov., isolated from rice roots. Int J Syst Evol Microbiol 66:3890–3896. doi:10.1099/ijsem.0.001284. 27393690

[4] Menezes CB, Tonin MF, Corrêa DB, Parma M, de Melo IS, Zucchi TD, Destéfano SA, Fantinatti-Garboggini F 2015 *Chromobacterium amazonense* sp. nov. isolated from water samples from the Rio Negro, Amazon, Brazil. Antonie van Leeuwenhoek 107:1057–1063. doi:10.1007/s10482-015-0397-3. 25663027

[5] Soby SD, Gadagkar SR, Contreras C, Caruso FL 2013 *Chromobacterium vaccinii* sp. nov., isolated from native and cultivated cranberry (*Vaccinium macrocarpon* Ait.) bogs and irrigation ponds. Int J Syst Evol Microbiol 63:1840–1846. doi:10.1099/ijs.0.045161-0. 22984138

[6] Kämpfer P, Busse HJ, Scholz HC 2009 *Chromobacterium piscinae* sp. nov. and *Chromobacterium pseudoviolaceum* sp. nov., from environmental samples. Int J Syst Evol Microbiol 59:2486–2490. doi:10.1099/ijs.0.008888-0. 19622653

[7] Han XY, Han FS, Segal J 2008 *Chromobacterium haemolyticum* sp. nov., a strongly haemolytic species. Int J Syst Evol Microbiol 58:1398–1403. doi:10.1099/ijs.0.64681-0. 18523185

[8] Martin PAW, Gundersen-Rindal D, Blackburn M, Buyer J 2007 *Chromobacterium subtsugae* sp. nov., a betaproteobacterium toxic to Colorado potato beetle and other insect pests. Int J Syst Evol Microbiol 57:993–999. doi:10.1099/ijs.0.64611-0. 17473247

[9] Meier-Kolthoff JP, Auch AF, Klenk HP, Göker M 2013 GBDP on the grid: a genome-based approach for species delimitation adjusted for an automated and highly parallel processing of large data sets. Hochleistungsrechnen in Baden-Württemberg—Ausgewählte Aktivitäten im bwGRiD 2012. KIT Scientific Publishing, Karlsruhe, Germany.

[10] Meier-Kolthoff JP, Auch AF, Klenk HP, Göker M 2013 Genome sequence-based species delimitation with confidence intervals and improved distance functions. BMC Bioinformatics 14:60. doi:10.1186/1471-2105-14-60. 23432962 PMC3665452

[11] Lima-Bittencourt CI, Astolfi-Filho S, Chartone-Souza E, Santos FR, Nascimento AMA 2007 Analysis of *Chromobacterium* sp. natural isolates from different Brazilian ecosystems. BMC Microbiol 7:58. doi:10.1186/1471-2180-7-58. 17584942 PMC1913517

